# Morphological Changes of Frontal Areas in Male Individuals With HIV: A Deformation-Based Morphometry Analysis

**DOI:** 10.3389/fneur.2022.909437

**Published:** 2022-06-27

**Authors:** Guochao Chen, Dan-Chao Cai, Fengxiang Song, Yi Zhan, Lei Wei, Chunzi Shi, He Wang, Yuxin Shi

**Affiliations:** ^1^Shanghai Institute of Medical Imaging, Fudan University, Shanghai, China; ^2^Shanghai Public Health Clinical Center, Fudan University, Shanghai, China; ^3^Institute of Science and Technology for Brain-Inspired Intelligence, Fudan University, Shanghai, China; ^4^Human Phenome Institute, Fudan University, Shanghai, China

**Keywords:** HIV, MRI, deformation-based morphometry, functional connectivity, cognitive assessment

## Abstract

**Objective:**

Previous studies on HIV-infected (HIV+) individuals have revealed brain structural alterations underlying HIV-associated neurocognitive disorders. Most studies have adopted the widely used voxel-based morphological analysis of T1-weighted images or tracked-based analysis of diffusion tensor images. In this study, we investigated the HIV-related morphological changes using the deformation-based morphometry (DBM) analysis of T1-weighted images, which is another useful tool with high regional sensitivity.

**Materials and Methods:**

A total of 157 HIV+ (34.7 ± 8.5 years old) and 110 age-matched HIV-uninfected (HIV-) (33.7 ± 10.1 years old) men were recruited. All participants underwent neurocognitive assessments and brain scans, including high-resolution structural imaging and resting-state functional imaging. Structural alterations in HIV+ individuals were analyzed using DBM. Functional brain networks connected to the deformed regions were further investigated in a seed-based connectivity analysis. The correlations between imaging and cognitive or clinical measures were examined.

**Results:**

The DBM analysis revealed decreased values (i.e., tissue atrophy) in the bilateral frontal regions in the HIV+ group, including bilateral superior frontal gyrus, left middle frontal gyrus, and their neighboring white matter tract, superior corona radiata. The functional connectivity between the right superior frontal gyrus and the right inferior temporal region was enhanced in the HIV+ group, the connectivity strength of which was significantly correlated with the global deficit scores (*r* = 0.214, *P* = 0.034), and deficits in learning (*r* = 0.246, *P* = 0.014) and recall (*r* = 0.218, *P* = 0.031). Increased DBM indexes (i.e., tissue enlargement) of the right cerebellum were also observed in the HIV+ group.

**Conclusion:**

The current study revealed both gray and white matter volume changes in frontal regions and cerebellum in HIV+ individuals using DBM, complementing previous voxel-based morphological studies. Structural alterations were not limited to the local regions but were accompanied by disrupted functional connectivity between them and other relevant regions. Disruptions in neural networks were associated with cognitive performance, which may be related to HIV-associated neurocognitive disorders.

## Introduction

People living with HIV, if left untreated, can progress to acquired immunodeficiency syndrome. Combined antiretroviral therapy can effectively control virus replication but cannot control the intracranial virus infection due to the blood-brain barrier, leading to central nervous system complications, especially the HIV-associated neurocognitive disorders (HAND). The pathogenesis of HAND is unclear, which could be attributed to the stimulation of macrophages and glial cells; these cells release cytokines and chemokines to damage neurons (1–3). The incidence rate of HAND is about 10–50%, even up to 69%, according to previous studies (4–6). The life quality and compliance with drug treatment of the HIV-infected (HIV+) individuals could be severely affected as HAND develops (7). Therefore, the early detection of HAND is beneficial to clinical treatment. Yet, the current HAND diagnostic criteria are purely based on lengthy neuropsychological tests, without input from other methods, such as brain imaging. As a non-invasive imaging technique with high resolution, magnetic resonance imaging (MRI) is an optimal method to reveal the brain structural and functional alterations in the HIV+ individuals, which can aid the HAND diagnosis at the preclinical or early stage.

Voxel-based morphometry (VBM) analysis of T1-weighted images is one of the most commonly used methods for analyzing brain morphology based on structural images (8). Previous VBM studies on HIV+ individuals have found structural alterations mainly in gray matter (9–12). Li et al. conducted VBM analysis on HIV+ men with normal cognition and HIV-uninfected (HIV-) healthy controls (9). Decreased volumes were found in the HIV+ group in the left superior temporal gyrus, cingulate gyrus, and a large portion of frontal areas, including the triangular and orbital part of the left inferior frontal gyrus (IFG) and right middle frontal gyrus (MFG); increased volumes were found around the midbrain aqueduct and lateral ventricle. Wang et al. also found decreased gray matter volume in the frontal cortex, including bilateral superior frontal gyrus (SFG), left MFG, and left IFG, together with decreased volume in the bilateral anterior cingulate cortex and left the supplementary motor area in HIV+ individuals (10). When HIV+ individuals with cognitive impairment were compared to HIV- controls, decreased gray matter volume was found mostly in the temporal cortex and anterior cingulate gyrus (13).

White matter volume changes in HIV+ individuals were also reported in other VBM studies (14, 15). Sarma et al. demonstrated that perinatal HIV infection could lead to structural abnormalities in both the white and gray matter: in addition to gray matter volume increase in left SFG, bilateral inferior and middle temporal gyrus, parahippocampal gyrus, and other brain regions, there was white matter atrophy in ventral temporal areas, bilateral posterior corpus callosum, external capsule, mid cerebral peduncles, and basal pons (14). Sanford et al. found that volume reductions in HIV+ subjects occurred in the white matter of the brainstem and thalamus, but not in gray matter or cerebrospinal fluid (14). In sum, previous MRI studies using VBM analysis have revealed both atrophy and enlargement of different brain areas in HIV+ individuals, which mainly occur in the gray matter of frontal, temporal, and cingulate cortexes.

Although less frequently reported than gray matter alterations in VBM analysis of T1-weighted images, HIV-associated white matter alterations have been widely revealed *via* tract-based spatial statistics analysis in diffusion tensor imaging (DTI) studies (10, 16–19). Wang et al. observed decreases in fractional anisotropy (FA) in the corpus callosum and anterior corona radiata and increases in mean diffusivity (MD), radial diffusivity (RD), and axial diffusivity (AD) in most skeleton locations at the early stage of HIV infection (10). Kuhn et al. also revealed that HIV infection was significantly related to lower FA and higher MD, AD, and RD across all regions (18).

Deformation-based morphometry (DBM) is another useful method to explore morphological changes in T1-weighted structural images, which analyzes the displacement between each voxel and the standard brain (20). Unlike VBM, DBM does not rely on automatic segmentation of gray matter, white matter, and cerebrospinal fluid. It matches local images based on the similarity of contrast and intensity using a robust nonlinear image registration algorithm and is thus capable of detecting boundaries and deformations (21–23). DBM analyses have been applied to many neurological diseases, including Alzheimer's disease, epilepsy, and schizophrenia, to reveal morphological changes in the brain (21, 24, 25). However, a thorough search of DBM analysis on HIV+ people yielded only a few studies (15, 26, 27). Kuhn et al. found a significant HIV effect on subcortical structures, including left putamen, right caudate, right nucleus accumbens, and bilateral hippocampus (26). Becker et al. estimated the DBM indexes in the caudate nucleus and putamen of HIV+ subjects, and significant atrophy was found in both regions (27). Sanford et al. used both VBM and DBM to compare the structural images of 125 HIV+ and 62 HIV- individuals (15). While the VBM analysis suggested white matter volume reduction in the brainstem and thalamus in the HIV+ group, the DBM analysis found no significant group differences, although similar brain regions to that of VBM were revealed when a lenient statistical threshold was used.

Furthermore, MRI studies on HIV+ participants reported HIV-associated brain functional abnormalities. In a study on HIV+ individuals with asymptomatic HAND, the reduced functional connectivity within the default mode network was found compared to the HIV- group (28). Similarly, Ann et al. investigated the resting-state default mode network in HIV+ subjects and found that the functional connections between the bilateral precuneus and prefrontal cortex, specifically the right IFG and SFG, were reduced in HAND patients compared to HIV+ individuals without HAND (29). Other intrinsic resting-state networks, including cortical-striatal and frontostriatal networks, were also found disrupted in HIV+ compared to HIV- individuals (30, 31). However, few studies have focused on the functional abnormalities of the morphologically altered brain areas (32). One recent study found that HIV+ individuals suffered from brain atrophy in brain regions within the visual network, and the functional connection between these regions and the default mode network was also altered (32).

To summarize, previous imaging studies on HIV+ individuals mainly focused on VBM analysis of T1-weighted images and tract-based analysis of diffusion-based images. HIV-associated brain structural alterations were mostly found in the gray matter based on T1-weighted images while white matter changes were reported in diffusion-based imaging studies. However, the functional abnormality in these altered brain regions remains to be investigated. The current study aimed to explore the HIV-associated brain structural changes in T1-weighted images *via* DBM analysis, which was sensitive to local morphological deformation. Functional connectivity between the deformed brain regions and the rest of the brain was also explored based on resting-state functional MRI images. Finally, associations of altered imaging indexes with cognitive performances and clinical indexes were also examined.

## Materials and Methods

### Participants

This study has been reviewed and approved by the ethics committee of the Shanghai Public Health Clinical Center in Shanghai, China. All participants were screened in strict accordance with the inclusion and exclusion criteria and gave their informed consent. The inclusion criteria were as follows: ① 18–60 years old; ② right-handedness; ③ HIV seropositive. The exclusion criteria were as follows: ① previous non-HIV-related acute and chronic neurological diseases, such as multiple sclerosis, Parkinson's disease, previous stroke, and neurosyphilis; ② history of organic central nervous system infection or neoplasm; ③ traumatic brain injury with loss of consciousness lasting more than 30 min; ④ mental diseases, such as schizophrenia, depression, and anxiety; ⑤ history of drug or alcohol dependence; ⑥ MRI contraindications; ⑦ color-blindness or color-weakness. The clinical data of HIV+ individuals, including current CD4 and CD8 cell counts, nadir CD4 and CD8 cell counts, current viral load, historical highest viral load, duration of HIV diagnosis, and duration of antiretroviral treatment, were collected from clinical records.

### Neuropsychological Tests

All the participants completed the systematic neurocognitive tests according to the Frascati standard (4), which evaluated cognitive abilities in the following seven domains: ① information processing speed [Wechsler adult intelligence scale-III (WAIS-III) digit symbol and WAIS-III symbol search]; ②, ③ learning and memory [Hopkins verbal learning test-revised (HVLT-R) and brief visuospatial memory test-revised (BVMT-R); ④ abstract/executive functions [Wisconsin card sorting test 64-card version (WCST-64) and category test]; ⑤ verbal fluency [category fluency (animals and actions)]; ⑥ attention/working memory [Wechsler memory scale-III (WMS-III) spatial span and paced auditory serial addition test (PASAT)]; ⑦ motor (grooved pegboard Test). Raw scores of tests were converted to demographically corrected standard scores (T scores), which were then converted to deficit scores from 0 (no impairment, T > 40) to 5 (severe impairment, T ≤ 20). Domain deficit scores were calculated by averaging the deficit scores of tests within each cognitive domain. The global deficit score (GDS) was calculated by averaging domain deficit scores to determine the overall cognitive impairment. GDS ≥0.5 indicates that the individual meets the clinical diagnosis of cognitive impairment (33).

### MRI Data Acquisition

Philips Ingenia 3T (Netherlands) and Siemens Skyra 3T (Germany) MRI systems were used in this study. The subjects were in the supine position in the scanner, wearing earplugs and headphones, and were indicated to keep quiet and avoid head movement during the scan. They were further required to keep their eyes closed and remain awake during the resting-state scanning. T1-weighted sequence was used for structural image scanning (Philips: TR = 8.2 ms, TE = 3.8 ms, FA = 8°, FOV = 240 × 240, voxel size = 1 × 1 × 1 mm^3^, slice number = 170; Siemens: TR = 1,900 ms, TE = 3.4 ms, FA = 9°, FOV = 250 × 250, voxel size = 1 × 1 × 1 mm^3^, slice number = 192). Resting-state functional imaging adopts gradient-echo single-shot echo-planar imaging (EPI) sequence (Philips: TR = 2,000 ms, TE = 25 ms, FA = 75°, FOV = 224 × 224, slice number = 35, slice thickness = 4 mm, number of volumes = 240; Siemens: TR = 2,500 ms, TE = 30 ms, FA = 75°, FOV = 225 × 225, slice number = 41, slice thickness = 3.5 mm, number of volumes = 240). In addition, conventional T2WI, FLAIR, and DWI imaging data were collected to evaluate whether abnormal signals or organic lesions were present in the brain. Two subjects were excluded due to head motion and metal artifacts in structural image scanning.

### MRI Data Analysis

Deformation-based morphometry analysis was performed using the SPM toolkit CAT12 (https://dbm.neuro.uni-jena.de/cat) and default parameters. According to the deformation field generated by the standardization process, the Jacobian determinant could be calculated, which corresponds to the deformation information of each voxel. The results are superimposed on the Mori atlas (http://www.neuro.uni-jena.de/cat12-html/cat_methods_RBM.html#Mori), which includes both gray matter and white matter structures. The SPM toolkit DPABI (http://rfmri.org/dpabi, v4.2) was used to preprocess the resting-state functional images. The standard preprocessing steps included slice timing correction, realignment, co-registration to structural images, spatial normalization, and spatial smoothness. The preprocessed data were resampled into 3 mm isotropic voxels and spatially smoothed with a 6 mm full width at half-maximum (FWHM) isotropic Gaussian kernel. Artifact Detection Tools (http://www.nitrc.org/projects/artifact_detect) and the default settings were used to identify problematic volumes in each scan. Specifically, a volume was defined as an outlier if the scan-to-scan head-motion composite changes were >2 mm, or if the *Z*-score of scan-to-scan global signal change was >9. Scans with more than 10 outlier volumes were excluded from the subsequent analysis. Mean framewise displacement during the scan was calculated for each participant to represent the extent of head motion. According to the DBM analysis, brain regions with significant structural changes in HIV+ people were identified. To explore the changes in the brain functional networks related to local brain structural changes, we defined these areas as regions of interest (ROIs) and further analyzed their functional connection with the whole brain. We did not take the cerebellar region as one of the ROIs because it was not fully covered in the resting-state functional scanning for most subjects.

### Statistical Analysis

The group comparisons of demographics, clinical information, neurocognitive performance, and head motion parameters were carried out using two-sample *t*-tests or Wilcoxon rank-sum tests depending on whether or not the variables were normally distributed (Shapiro-Wilk tests, *P* < 0.05). Age, years of education, and machine model were considered as covariates to reduce the potential effects in statistical analyses on DBM and brain network measures. Family-wise error (FWE) correction was applied to correct multiple comparisons with statistical significance defined by voxel-level *P* < 0.001 and cluster level *P* < 0.05. Correlation analyses were conducted to explore the relationships between the altered imaging characteristics and clinical and cognitive measures in the HIV+ group. Clinical measures included current CD4 and CD8 cell count, nadir CD4 and CD8 cell count, historical highest viral load, duration of HIV diagnosis, and duration of antiretroviral treatment; the current viral load was not included as the majority of HIV+ subjects showed undetectable viral loads. Cognitive measures included the GDS and deficit scores in seven cognitive domains. Shapiro-Wilk tests were used to test the normality of all measures. As all clinical and cognitive measures were not normally distributed, Spearman's correlation was used in the correlation analysis.

## Results

### Demographics

Table 1 summarizes the demographics and neuropsychological and clinical characteristics of the two groups of subjects. A total of 230 HIV+ and 182 HIV- individuals were enrolled in this study, among which 86.9% were males. To reduce the potential effect of age, education, and sex in the MRI measures, only male subjects with matched age and education years in both groups were included. The final analysis included 157 HIV+ men (mean age = 34.73 ± 8.46 years old, years of education = 14.43 ± 2.4 years) and 110 HIV- men (mean age = 33.67 ± 10.1 years old, years of education = 13.85 ± 2.63 years). Among them, 128 HIV+ and 84 HIV- participants were scanned on the Philips scanner, and the remaining 29 HIV+ and 26 HIV- participants were scanned on the Siemens scanner. No significant group differences were detected in age (*P* = 0.37), years of education (*P* = 0.07) and mean framewise displacement (*P* = 0.8). All the HIV+ people were treated with standard antiretroviral drugs. Among them, 79.34% were diagnosed more than 1 year ago, and 35.9% had the highest viral load of more than 50 copies/ml. Among the 109 with known transmission routes, male homosexual contact accounted for 84.4%. The current CD4 value was 471.11 ± 198.87 copies/mL, and the lowest historical CD4 value was 213.26 ± 141.73 copies/mL. In this study, 100 HIV+ and 47 HIV- individuals completed the standard neurocognitive assessment. According to the GDS standard, 21 HIV+ and 10 HIV- individuals were cognitively impaired. No significant group difference was detected in either the GDS or any of the domain deficit scores.

**Table 1:** Demographics, neuropsychological and clinical information.

		**HIV+**	**HIV-**	***P*** **-value**
N		157	110	
Machine	Siemens	128	84	
	Philips	29	26	
Age [mean ± SD], y		34.73 ± 8.46	33.67 ± 10.10	0.37
Education [mean ± SD], y		14.43 ± 2.40	13.85 ± 2.63	0.07
Duration of HIV diagnosis > 1 y, %		79.34%	NA	
Current CD4, copies/mL		471.11 ± 198.87	NA	
Nadir CD4, copies/mL		213.26 ± 141.73	NA	
Current CD8, copies/mL		839.42 ± 350.05	NA	
Sexual transmission, %		84.40%	NA	
Viral load (> 50 copies/mL), %		35.9%	NA	
GDS, N		0.34 ± 0.44, 100	0.29 ± 0.37, 47	0.40^a^
GDS ≥ 0.5		21	10	
Mean framewise displacement		0.14 ± 0.08	0.15 ± 0.10	0.80

a *P-value from the Wilcoxon rank-sum test*.

### MRI Results

**Figure 1:**
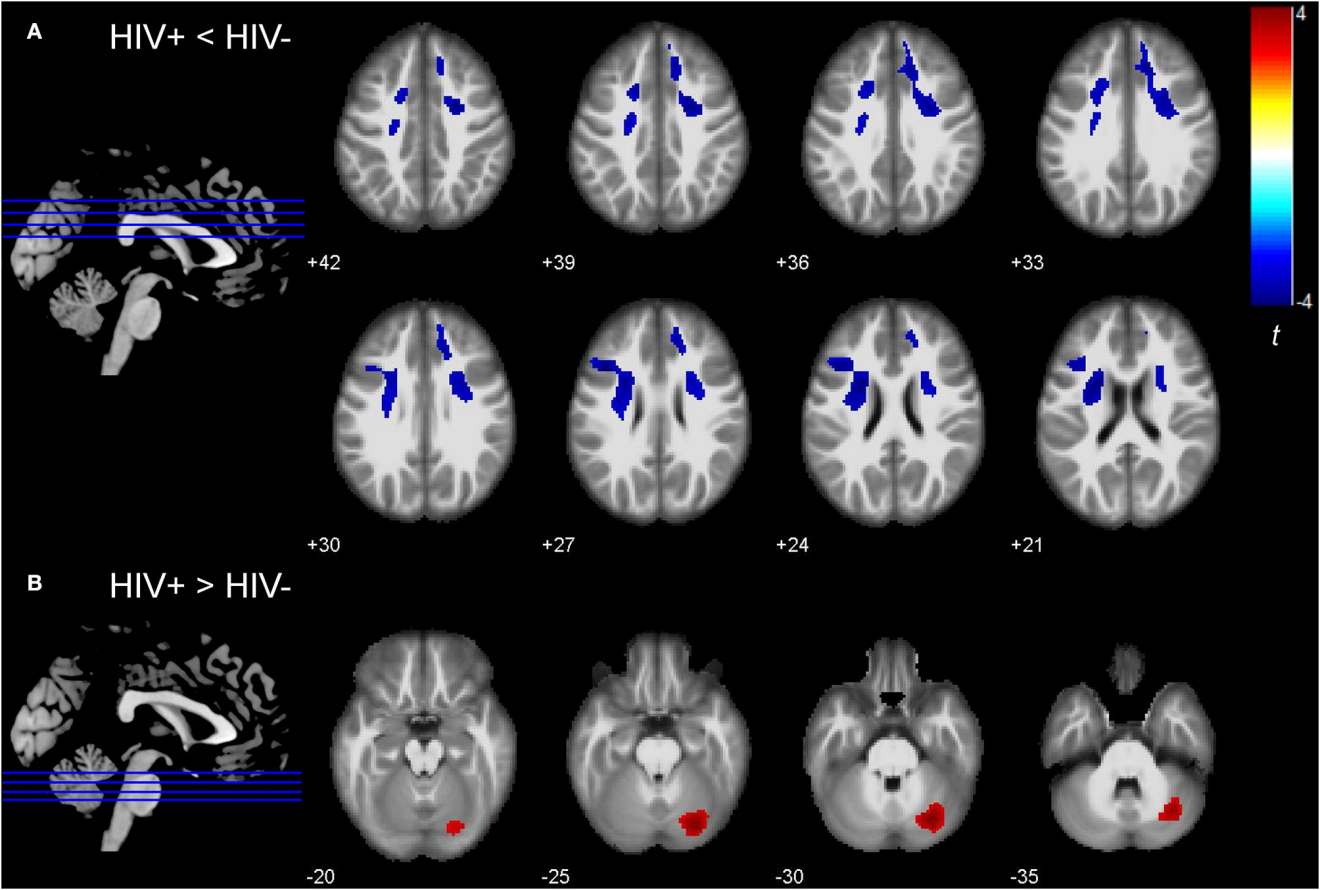
Group differences in deformation-based morphometry (DBM) indexes between HIV infected (HIV+) and HIV uninfected (HIV-) group. **(A)** Bilateral superior and left middle frontal regions showed decreased DBM indexes (i.e., tissue atrophy) in the HIV+ group (voxel-level uncorrected *P* < 0.001, cluster level *P* < 0.05, FWE corrected). **(B)** Right cerebellum showed increased DBM indexes (i.e., tissue enlargement) in the HIV+ group (voxel-level uncorrected *P* < 0.001, cluster level *P* < 0.05, FWE corrected). The colors indicate the *t*-statistics.

Deformation-based morphometry analysis suggested that, compared to the HIV- group, the HIV+ group showed tissue atrophy in the bilateral frontal regions, including bilateral SFG, left MFG, and their surrounding white matter tract, bilateral superior corona radiata (Figure 1A). Tissue expansion was found in the right cerebellum (Figure 1B). Table 2 shows the MNI coordinates of the altered brain regions (voxel-level uncorrected *P* < 0.001, cluster level *P* < 0.05, FWE corrected). Correlations between the deformation statistics in these brain regions are presented in Supplementary Table 1. No significant correlation was found between the DBM indexes and clinical or cognitive measures.

**Table 2:** Deformation differences between HIV+ and HIV- groups.

**Structure**		**L/R**	**Cluster size (mm^**3**^)**	**Coordinates**			**t value**	**P-value**
				***x***	***y***	***z***		
**HIV+ < HIV-**								
Cluster1	SFG	R	3,792	24	2	42	4.06	<0.001
	SCR	R	3,376	29	8	32	3.78	<0.001
Cluster2	SCR	L	4,088	−24	5	23	4.06	<0.001
	MFG	L	2,168	−42	20	26	4.04	<0.001
	SFG	L	1,352	−18	15	38	3.73	<0.001
**HIV+ > HIV-**								
Cerebellum		R	5,272	29	−75	−29	4.19	<0.001

*SFG, superior frontal gyrus; SCR, superior corona radiata; MFG, middle frontal gyrus; L, left; R, right*.

**Figure 2:**
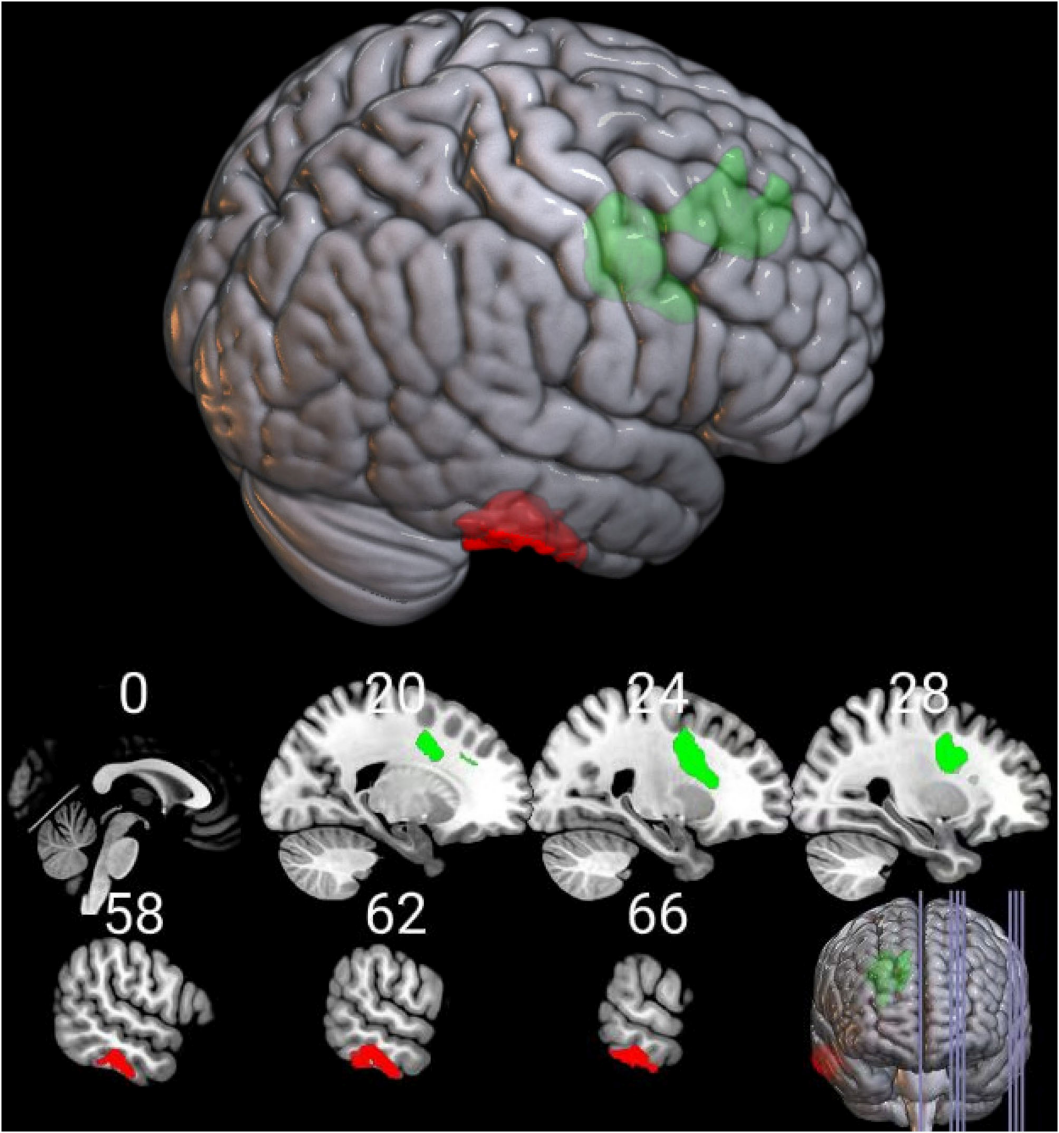
Functional alterations of the deformed brain region in the HIV+ group. Functional connection between the deformed right superior frontal region (in green color) and the right inferior temporal gyrus (in red color) was significantly increased in the HIV+ group (voxel-level uncorrected *P* < 0.005, cluster level *P* < 0.05, FWE corrected).

**Figure 3:**
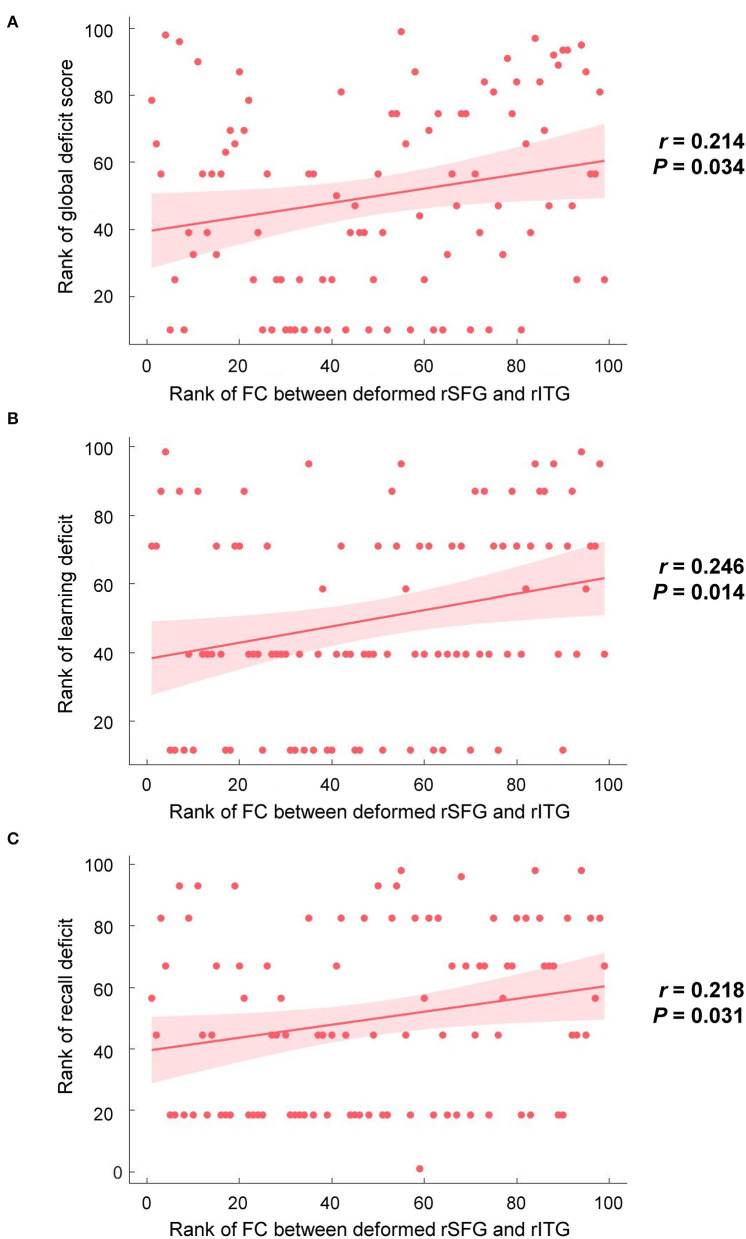
Correlations between brain alterations and cognitive performances in the HIV+ group. **(A)** The functional connection (FC) strength between the deformed right superior frontal gyrus (rSFG) and the right inferior temporal gyrus (rITG) was positively correlated with global deficit scores (*r* = 0.214, *P* = 0.034). **(B)** The FC strength between the deformed rSFG and rITG was positively correlated with the deficit score of learning (*r* = 0.246, *P* = 0.014). **(C**) The FC strength between the deformed rSFG and rITG was positively correlated with the deficit score of recall (*r* = 0.218, *P* = 0.031).

Brain regions with significant group differences in the DBM analysis were defined as ROIs. ROI-based functional connectivity analysis was conducted in 154 HIV+ and 104 HIV- individuals after nine subjects were excluded due to excessive head motion. The results showed enhanced functional connectivity between the right superior frontal region and the right inferior temporal gyrus (Figure 2). The connectivity strength between the above two brain regions was positively correlated with the GDS (Figure 3A, *r* = 0.214, *P* = 0.034), learning deficits (Figure 3B, *r* = 0.246, *P* = 0.014) and recall deficits (Figure 3C, *r* = 0.218, *P* = 0.031). No significant correlation was found between connectivity strength and clinical measures.

## Discussion

In this study, DBM was used to analyze the brain structural changes of HIV+ people. Furthermore, dysfunctional connections between the deformed brain areas and the rest of the brain were explored *via* seed-based analyses. The associations of brain imaging measures with clinical indexes and cognitive impairments were also examined. The present study demonstrated that, compared to the HIV- group, the HIV+ group had decreased volume in the bilateral superior frontal lobe and increased volume in the right cerebellum. More importantly, the functional connection between the atrophic right superior frontal region and the right inferior temporal gyrus was enhanced, which was further related to GDS and deficits in learning and recall. These results showed that there were detectable brain structural and functional changes in HIV+ people which may be the underlying brain mechanism of HAND.

One of the main findings of the current study was the tissue atrophy in the white matter tract of superior corona radiata, which connects to the neighboring gray matter. The deformation statistics between the white matter tract and the neighboring gray matter regions were highly correlated (correlation coefficients larger than 0.5, Supplementary Table 1), leading to the assumption that deformation of the superior corona radiata might mediate the observed group differences in neighboring gray matter regions. Deformation in this white matter tract was consistent with previous HIV studies based on DTI, which is a different imaging technique from the T1-weighted structural imaging used in the current study. One study showed that, compared with HIV- individuals, HAND patients had a significantly increased MD in the widespread white matter, including the bilateral superior and posterior corona radiata (17). Another study also showed that HIV+ patients exhibited significantly higher MD and increased AD in the bilateral superior corona radiata and other white matter regions (16). However, we did not find white matter alterations in the brainstem and thalamus, which were revealed by a previous DBM study on HIV+ individuals, although not statistically significant (15). Nonetheless, the current finding of atrophic superior corona radiata in the HIV+ group supported the HIV-associated pathophysiological changes in white matter (19, 34).

Besides bilateral superior corona radiata, deformation of gray matter in the neighboring frontal areas, including bilateral SFG and left MFG, was also revealed by the DBM analysis, which was consistent with previous VBM studies (9, 13, 35, 36). Specifically, HIV+ individuals had significantly less gray matter than the HIV- group in the medial and superior frontal gyrus (37). A meta-analysis showed that the frontal cortex was the brain region most frequently affected by HIV infection, especially in HIV+ people with normal cognition, suggesting an early HIV effect on the frontal cortex (38). In the current study, most people did not show any obvious neurocognitive impairment at the behavioral level, with only 21% of the HIV+ subjects having an asymptomatic impairment. The overall better cognitive performance in the HIV+ individuals compared to previous studies might explain why brain atrophy was only found in the frontal regions in the current study. However, we did not find HIV-related deformation in subcortical structures as reported by previous studies (26, 27, 39). One reason for this discrepancy might be that a whole-brain analysis was conducted in the current study while previous studies focused on specific subcortical regions.

The HIV-related brain changes were not limited to the deformed frontal regions. Based on the seed-based analysis of resting-state fMRI data, we found that the functional connection of the deformed superior frontal region with the right inferior temporal gyrus was enhanced in the HIV+ group. The connectivity enhancement between these two regions might indicate compensatory activity outside the deformed regions in HIV+ individuals. This is in accordance with previous studies showing the impact of HIV infection on the temporal lobe (13, 40). Previous studies have shown that brain injury reduces neural efficiency and requires more neural resources to maintain cognitive function, which is called cognitive reserve (41, 42). Some fMRI studies revealed that HIV+ patients showed greater brain activation in the frontal lobes when performing tasks, indicating that injury to the frontal lobes caused by HIV needs greater modulation than the use of the brain reserve (43–47). Further studies are needed to explore the network connectivity and compensatory activities between the frontal and temporal lobes.

Furthermore, we found that the dysconnectivity between the deformed right frontal region and inferior temporal gryus was associated with GDS, learning deficits, and recall deficits. A recent review summarized that it is partially induced by subtle synaptodendritic damage and disruption of neuronal networks in brain areas that mediate learning and memory (48). Similar findings in HIV+ individuals have been reported by Ann and her colleagues (29). They found that the connectivity strength between right frontal areas, which included right SFG and IFG and are highly overlapped with our DBM finding, and bilateral precuneus was positively correlated with the deficit score of delayed recall memory. It is possible that the white matter within the deformed frontal areas (i.e., the superior corona radiata) plays an important role in delayed recall memory (16, 49). Li et al. employed the same memory test as in the current study to investigate the learning and recall ability of HIV+ people (16). They found that memory scores were negatively correlated with the MD in the right superior corona radiata. Similarly, Hoare et al. showed that HIV+ participants with poor prospective memory had significantly decreased anisotropic fraction in superior corona radiata (49). Altogether, these correlations between abnormal imaging measures and neuropsychological declines in HIV+ individuals supported the potential value of imaging markers in HAND diagnosis.

In addition to the structural changes in the superior and middle frontal lobes, DBM also found increased volume in the right cerebellum in the HIV+ group. Previous VBM studies on HIV+ individuals reported both atrophy (50–52) and enlargement (53) in the cerebellum, depending on the severity of central nervous system damage. Increased cerebellar volume may reflect the proliferation of macrophages and glial cells caused by persistent HIV-related neuro-inflammation, resulting in the swelling of brain cells and tissues. In that case, the enlarged cerebellum is a sign of neurological progression from asymptomatic to symptomatic HAND (1). Another explanation was that the increase in cerebellar volume may be a compensatory change after brain parenchymal lesions are induced by HIV infection (53).

There are several limitations in the present study. First, two scanners were used to collect the data. Although the scanner was treated as a covariate in statistical analysis, the impact of different equipment cannot be fully excluded. Second, this was a cross-sectional study. Further longitudinal analysis is needed to verify the relationship between brain functional changes and cognitive performance. Third, it was not clear whether different analytical methods might lead to substantially different findings. We did not conduct other morphological analyses, such as VBM and surface-based morphometry, in the current study, as our objective was to complement previous HIV imaging studies by focusing on DBM. According to a previous study using both VBM and DBM analyses, VBM detected significant brain alterations in HIV+ subjects while DBM did not, although the findings from both methods were similar when a lenient statistical threshold was applied (15). Future HIV imaging studies with larger sample sizes were needed to investigate the methodological advantages and disadvantages.

## Conclusion

The current study employed DBM analysis on HIV+ individuals and revealed tissue atrophy in the frontal cortex, including bilateral SFG, left MFG, and their neighboring white matter tract of superior corona radiata, and also revealed tissue enlargement in the right cerebellum. Structural alterations were not limited to local changes but were accompanied by disrupted functional connectivity between deformed regions and other relevant regions. Disruptions in brain anatomy or neural networks were associated with deficits in overall cognitive performance, especially in learning and recall. These results indicate the potential value of DBM analysis in assessing HIV-associated brain alterations in T1-weighted images in addition to VBM.

## Data Availability Statement

The raw data supporting the conclusions of this article will be made available by the authors, without undue reservation.

## Ethics Statement

The studies involving human participants were reviewed and approved by Ethics Committee of the Shanghai Public Health Clinical Center in Shanghai, China. The patients/participants provided their written informed consent to participate in this study.

## Author Contributions

GC and D-CC: study design, participant recruitment, data collection, data analysis, and manuscript writing. FS: participant recruitment and data collection. YZ and CS: participant recruitment. LW: data analysis. HW: study conceptualization. YS: study design, study conceptualization, and project administration. All authors were involved in interpreting the results and modifying the manuscript.

## Funding

This work was supported by the Shanghai Municipal Commission of Health and Family Planning (201840146), the Shanghai Municipal Health Commission (20204Y0334), the Science and Technology Commission of Shanghai Municipality (19411965800), and the National Institutes of Health grant from the National Institute of Neurological Disorders and Stroke (R01NS108809).

## Conflict of Interest

The authors declare that the research was conducted in the absence of any commercial or financial relationships that could be construed as a potential conflict of interest.

## Publisher's Note

All claims expressed in this article are solely those of the authors and do not necessarily represent those of their affiliated organizations, or those of the publisher, the editors and the reviewers. Any product that may be evaluated in this article, or claim that may be made by its manufacturer, is not guaranteed or endorsed by the publisher.
