# Supplementary material for: Morphological Changes of Frontal Areas in Male Individuals With HIV: A Deformation-Based Morphometry Analysis
Source: Front Neurol. 2022 Jun 27;13:909437. doi: 10.3389/fneur.2022.909437 (PMC9271794; doi:10.3389/fneur.2022.909437)
Supplement: Supplementary file 1 [file Table_1.DOCX]

Supplementary Material

# Supplementary Tables

**Table S1 |** Correlation between the deformation statistics of deformed clusters

|  |  |  | *Cluster1* |  |  | *Cluster2* |  |  |  | *Cerebellum* |
| --- | --- | --- | --- | --- | --- | --- | --- | --- | --- | --- |
|  |  |  |  | R | R |  | L | L | L | R |
|  |  |  |  | SFG | SCR |  | SCR | MFG | SFG |  |
| *Cluster1* |  |  |  |  |  | *0.879* |  |  |  | *0.303* |
|  | R | SFG | 0.933 |  |  |  |  |  |  |  |
|  | R | SCR | 0.876 | **0.644** |  |  |  |  |  |  |
| *Cluster2* |  |  |  |  |  |  |  |  |  | *0.267* |
|  | L | SCR |  | 0.635 | 0.936 | 0.924 |  |  |  |  |
|  | L | MFG |  | 0.641 | 0.603 | 0.850 | **0.591** |  |  |  |
|  | L | SFG |  | 0.694 | 0.773 | 0.804 | **0.757** | 0.582 |  |  |
| *Cerebellum* | R |  |  | 0.299 | 0.243 |  | 0.239 | 0.246 | 0.182 |  |

SFG: superior frontal gyrus; SCR: superior corona radiata; MFG: middle frontal gyrus. Pearson’s correlation coefficients are presented. Bold indicates the correlation between the white matter tract of SCR and gray matter regions. Italic indicates correlation between clusters.
